# A new horsehair worm, *Chordodes formosanus* sp. n. (Nematomorpha, Gordiida) from *Hierodula* mantids of Taiwan and Japan with redescription of a closely related species, *Chordodes japonensis*

**DOI:** 10.3897/zookeys.160.2290

**Published:** 2011-12-29

**Authors:** Ming-Chung Chiu, Chin-Gi Huang, Wen-Jer Wu, Shiuh-Feng Shiao

**Affiliations:** 1 Department of Entomology, National Taiwan University, Taipei, Taiwan

**Keywords:** Nematomorpha, *Chordodes formosanus*, *C. japonensis*, new species, molecular analysis, immature stages, mantid hosts

## Abstract

A new species of horsehair worm, *Chordodes formosanus* **sp. n.**, is described and compared to a closely related species, *Chordodes japonensis*. Although both species possess the same six cuticular structures of areoles on the surface, the significantly longer filaments on the female crowned areoles can be used as diagnostic characters for the new species. The different taxonomic status of these two species was also confirmed after analyzing the partial cytochrome oxidase subunit I sequence, and the mantid hosts, which are respectively limited to the genus *Tenodera* for *Chordodes japonensis* and *Hierodula* for *Chordodes formosanus* **sp. n.** In addition, the immature stages of eggs and larvae of the new species are also described and discussed in detail.

## Introduction

The host range of an organism is an important ecological character since it reflects the survival and reproduction of parasites (Onstad and McManus 1996). Parasite taxonomy can be clear only when the taxonomic status of both the parasites and their hosts are well understood. Horsehair worms are obligate parasites that pass through different hosts at various stages (Hanelt et al. 2005). Among the 350 described species of horsehair worms in 21 genera (Schmidt-Rhaesa and Lalramliana 2011), the genus *Chordodes* consists of about 90 species, and is one of the most diverse genera in the phylum Nematomorpha (Schmidt-Rhaesa et al. 2008). Members of *Chordodes* can be easily distinguished from those of other genera by their unique cuticular structures known as crowned areoles (Schmidt-Rhaesa 2002a). Nevertheless, the various structures of the areoles are not always clear at the species level.

*Chordodes formosanus* sp. n. which is morphologically similar to *Chordodes japonensis* is hereby described as new to science*. Chordodes japonensis* was originally described by Inoue (1952) using light microscopy from specimens collected in Honsyu, Japan. At that time, this horsehair worm was known to parasitize the mantids *Tenodera sinensis* and *Tenodera angustipennis*. Following that, Baek (1993) checked horsehair worms specimens deposited in Kon-Kuk University, Seoul, Korea (collected from Kyongsangbuk-do, Seoul, Chollabuk-do, and Kyunggi-do, Korea) and identified them as *Chordodes japonensis* using scanning electron microscopy (SEM). Schmidt-Rhaesa (2004) checked horsehair worms (collected in Shiga, Japan) deposited in the Lake Biwa Museum, Shiga Prefecture, Japan. One female parasitized the mantid *Hierodula patellifera*, and one free-living male was considered to be *Chordodes japonensis* after examining it with SEM. Although the descriptions in the previous three papers slightly differ (see Table 1 in Schmidt-Rhaesa 2004), those authors believed the similar morphologies still would not make them clearly separated species. In other words, *Chordodes japonensis* was reported to be distributed in Japan and Korea and to have at least three mantid hosts, *Tenodera sinensis*, *Tenodera angustipennis*, and *Hierodula patellifera*.

However, in April 2007 to February 2008, we conducted a field survey of horsehair worms in Taiwan and found that the mantids, *Hierodula patellifera* and *Hierodula formosana*, were infested by *Chordodes japonensis*, but there was none in the 109 individuals of *Tenodera sinensis* examined. This geographical difference in the host specificity of *Chordodes japonensis* in Taiwan and Japan raised a question as to the taxonomic status of horsehair worms from the above three mantids. In order to answer this question, we examined morphological characters with both light and scanning electron microscopes, and the phylogeny was reconstructed using the mitochondrial (mt)DNA cytochrome oxidase subunit I (mtDNA-COI) gene of 40 adult horsehair worms collected from the three mantids, *Tenodera sinensis*, *Hierodula patellifera*, and *Hierodula formosana*, in Taiwan and Japan. We believe that these horsehair worms actually consist of two distinct species: *Chordodes japonensis* Inoue, 1952 from *Tenodera sinensis* in Japan, and a new species from *Hierodula patellifera* and *Hierodula formosana* in Japan and Taiwan. This paper deals with the new species of *Chordodes formosanus* sp. n., and descriptions of its egg and larval morphologies are also provided.

**Table 1.** Specimen information examined in the present study

Host					Horsehair worms			
Species	Collecting date	Locality	Longitude and latitude	Collector	Species	Sex	GenBank no.	Deposition
*Hierodula formosan*^a1^/	10-VII-2008	Xindian, New Taipei City, Taiwan	24°56'58.62"N, 121°34'2.90"E	Ming-Chung Chiu	*Chordodes formosanus*	Male	HM044112	NTU
*‘'*	*‘'*	*‘'*	*‘'*	*‘'*	*‘'*	Female	HM044113	NTU
*‘'*	*‘'*	*‘'*	*‘'*	*‘'*	*‘'*	Female	HM044114	NTU
*‘'*	*‘'*	*‘'*	*‘'*	*‘'*	*‘'*	Female	HM044115	NTU
*Hierodula formosana*	12-VII-2008	Xindian, New Taipei City, Taiwan	24°56'58.62"N, 121°34'2.90"E	Ming-Chung Chiu	*Chordodes formosanus*	Female	HM044119	LBM
*Hierodula formosana*	20-VII-2008	Xindian, New Taipei City, Taiwan	24°56'58.62"N, 121°34'2.90"E	Ming-Chung Chiu	*Chordodes formosanus*	Male	HM044116	NMNS
*‘'*	*‘'*	*‘'*	*‘'*	*‘'*	*‘'*	Male	HM044104	NMNS
*‘'*	*‘'*	*‘'*	*‘'*	*‘'*	*‘'*	Female^2/^	**HM044105**	NMNS
*Hierodula formosana*	10-VII-2009	Shimen, New Taipei City, Taiwan	NA	Chun-Kai Wang	*Chordodes formosanus*	Male	HM044123	LBM
*Hierodula formosana*	2-VIII-2007	Taipei Zoo, Taipei City, Taiwan	24°59'44.70"N, 121°34'49.49"E	Ming-Chung Chiu	*Chordodes formosanus*	Female	HQ322115	NTU
*‘'*	*‘'*	*‘'*	*‘'*	*‘'*	*‘'*	Female	HQ322116	NTU
*Hierodula formosana*^/3^	29-I-2008	Taipei Zoo, Taipei City, Taiwan	24°59'44.70"N, 121°34'49.49"E	Ming-Chung Chiu	*Chordodes formosanus*	Male	HM044122	NTU
*‘'*	*‘'*	*‘'*	*‘'*	*‘'*	*‘'*	Female	HM044121	NTU
*Hierodula formosana*	23-VII-2008	Jiaushi, Yilian, Taiwan	24°49'55.62"N, 121°44'50.12"E	Ming-Chung Chiu	*Chordodes formosanus*	Male	HM044111	NTU
*Hierodula formosana*	23-VII-2008	Jiaushi, Yilian, Taiwan	24°49'55.62"N, 121°44'50.12"E	Ming-Chung Chiu	*Chordodes formosanus*	Male	HM044118	LBM
*‘'*	*‘'*	*‘'*	*‘'*	*‘'*	*‘'*	Female	HM044108	LBM
*Hierodula formosana*	24-VI-2009	Jiaushi, Yilian, Taiwan	24°49'55.62"N, 121°44'50.12"E	Ming-Chung Chiu	*Chordodes formosanus*	Male^3/^	**HM044124**	NMNS
*‘'*	*‘'*	*‘'*	*‘'*	*‘'*	*‘'*	Male	HM044125	NMNS
*Hierodula formosana*	16-VII-2009	Jiaushi, Yilian, Taiwan	24°49'55.62"N, 121°44'50.12"E	Ming-Chung Chiu	*Chordodes formosanus*	Male	HM044126	NTU
*Hierodula formosana*	3-VIII-2009	Jiaushi, Yilian, Taiwan	24°49'55.62"N, 121°44'50.12"E	Ming-Chung Chiu	*Chordodes formosanus*	Male	HM044127	NMNS
*Hierodula formosana*	16-VII-2009	Jiaushi, Yilian, Taiwan	24°49'55.62"N, 121°44'50.12"E	Ming-Chung Chiu	*Chordodes formosanus*	Male	HM044128	NMNS
*Hierodula formosana*	23-VII-2008	Jiaushi, Yilian, Taiwan	24°49'55.62"N, 121°44'50.12"E	Ming-Chung Chiu	*Chordodes formosanus*	Female	HM044117	NMNS
*Hierodula formosana*	10-VII-2008	Jiaushi, Yilian, Taiwan	24°49'55.62"N, 121°44'50.12"E	Ming-Chung Chiu	*Chordodes formosanus*	Female	HM044120	NTU
*Hierodula formosana*	23-VII-2008	Jiaushi, Yilian, Taiwan	24°49'55.62"N, 121°44'50.12"E	Ming-Chung Chiu	*Chordodes formosanus*	Female	HM044106	NTU
*Hierodula formosana*	23-VII-2008	Jiaushi, Yilian, Taiwan	24°49'55.62"N, 121°44'50.12"E	Ming-Chung Chiu	*Chordodes formosanus*	Female	HM044109	NTU
*Hierodula formosana*	23-VII-2008	Jiaushi, Yilian, Taiwan	24°49'55.62"N, 121°44'50.12"E	Ming-Chung Chiu	*Chordodes formosanus*	Female	HM044110	NTU
*Hierodula formosana*	5-VII-2008	Taroko National Park, Hualien, Taiwan	NA	Tsung-Hung Yang	*Chordodes formosanus*	Male	HM044107	NTU
*Hierodula patellifera*	30-IX-2006	Taipei Zoo, Taipei City, Taiwan	24°59'44.70"N, 121°34'49.49"E	Ming-Chung Chiu	*Chordodes formosanus*	Male	JF808204	NTU
*Hierodula patellifera*	X-2008	Hsinchu City, Taiwan	NA	Ju-Chun Hsu	*Chordodes formosanus*	Female	JF808197	NTU
*Hierodula patellifer*^a3^/	18-VII-2003	Lyudao, Taitung, Taiwan	NA	Hsing-Yu Chou	*Chordodes formosanus*	Male	JF808203	NTU
*‘'*	*‘'*	*‘'*	*‘'*	*‘'*	*‘'*	Male	JF808205	NTU
*Hierodula patellifera*	16-X-2010	Sakado, Saitama, Japan	35°96'44.87N, 139°40'27.39E	Etsuko Suzuki	*Chordodes formosanus*	Female	JF808194	NTU
*Hierodula patellifera*	1-XI-2010	Kijo, Miyazaki, Japan	32°13'30.59"N, 131°24'16.62"E	Yasukuni Ono	*Chordodes formosanus*	Female	JF808198	LBM
*Hierodula patellifera*	1-XI-2010	Kijo, Miyazaki, Japan	32°13'30.59"N, 131°24'16.62"E	Yasukuni Ono	*Chordodes formosanus*	Female	JF808199	NTU
*Hierodula patellifera*	10-XI-2010	Kijo, Miyazaki, Japan	32°14'57.82"N, 131°23'3.93"E	Yasukuni Ono	*Chordodes formosanus*	Female	JF808202	NTU
*Hierodula patellifera*	11-XI-2010	Miyazaki, Miyazaki, Japan	31°56'54.15"N, 131°16'22.71"E	Yasukuni Ono	*Chordodes formosanus*	Female	JF808200	NTU
*Hierodula patellifera*	26-XI-2010	Kijo, Miyazaki, Japan	32°10'21.37"N, 131°27'36.53"E	Yasukuni Ono	*Chordodes formosanus*	Male	JF808196	NTU
*Hierodula patellifera*	26-XI-2010	Kijo, Miyazaki, Japan	32°12'55.36"N, 131°24'52.13"E	Yasukuni Ono	*Chordodes formosanus*	Female	JF808201	NTU
*Hierodula patellifera*	16-X-2010	Sakado, Saitama, Japan	5°96'44.87N, 139°40'27.39E	Wataru Toki	*Chordodes formosanus*	Female	JF808195	NTU
*Tenodera sinensis*	5-XI-2010	Kijo, Miyazaki, Japan	32°10'21.50"N, 131°27'36.53"E	Yasukuni Ono	*Chordodes japonensis*	Male	JF808206	NTU

LBM: Lake Biwa Museum; NMNS: National Museum of Natural Science; NTU: National Taiwan University. ^1^ No host specimen preserved. ^2 ^Allotype. ^3^ Holotype.

## Materials and methods

In total, the morphologies of 40 adult horsehair worms (including two females which laid eggs in the laboratory) were examined, and these worms were used for a DNA analysis. The morphologies of larvae laid in the laboratory were examined by light microscopy. Eggs and larvae collected in 2010 were examined by an SEM, and their COI sequences were analyzed to determine their taxonomic status. After studying the specimens, the partial bodies of these 40 samples were preserved in the Department of Entomology, National Taiwan University, Taipei; National Museum of Natural Science, Taichung, Taiwan; and Lake Biwa Museum, Shiga, Japan.

### Collection and preservation of horsehair worms

Mantids (*Hierodula formosana*, *Hierodula patellifera*, and *Tenodera sinensis*) infected with horsehair worms were collected from trees, shrubs, and grasses near water in Taiwan and Japan. Most of the adult horsehair worms emerged from the mantids after the hosts’ abdomens were immersed in water. Some individuals inside the mantids were only found after we had dissected the mantids. In total, 30 mantids with 40 horsehair worms inside them were examined (23 hosts with a single worm, five with two, one with three, and one with four, see Table 1). We first fixed the horsehair worms (except for two females which laid eggs (see below for detail)) and their hosts in a 75% alcohol solution for several days and then kept them in a 95% alcohol solution to preserve the DNA. Collection data are given in Table 1 including the locality, date, and collector.

Two pairs of adult horsehair worms (two males from Sindian, New Taipei City, and two females from Taipei Zoo) were collected on August 2, 2007. They were reared together in a plastic container (20 cm in diameter and 10.5 cm high) filled with 800 ml of aerated tap water, and maintained at 27 ± 1°C. The females were kept in water to lay eggs for 1 month, then fixed in a 75% alcohol solution and preserved in a 95% alcohol solution. Egg strings were found after 5 days and had hatched to larvae by 1 month later. Larvae were kept alive until being observed under light microscopy. Egg strings stuck on rocks were collected from Wufengqi Waterfalls, Yilan County, on July 21, 2010. They were brought back to the laboratory and kept in a tank with 20 L of tap water under the same conditions as described above. Eggs hatched about 8 weeks later and were then fixed and preserved as described above.

### Morphological examination

For adult specimens, the body surface was examined under light microscopy (Olympus BH-2, PM-10AD, Tokyo, Japan). For each specimen, a fragment of about 1 cm long of the mid-body was removed and cut longitudinally. Instead of using a scalpel to directly remove the internal tissues, we dipped the fragment into a 1% KOH solution for 2 h. The internal tissues became transparent and removable. The cuticles were placed on a microslide and observed under a microscope at a magnification of 40–200×. Eggs and newly hatched larvae were placed on the microslides, each with a drop of water and a cover glass. They were observed alive under the light microscope (at a magnification of 400×).

SEM was also used to examine adult and larval specimens. Its preparation protocol followed that of Schmidt-Rhaesa (2002b). Fragments of the anterior end, mid-body, and posterior end of preserved adult and larval specimens were dehydrated with a series of 75%, 95%, and 100% ethanol solutions and then replaced by acetone after using a series of alcohol/acetone mixtures of 2:1, 1:1, 1:2, and 0:1. Samples were then critical-point-dried, gold-sputter-coated, and examined under an SEM (JEOL JSM-5600, Tokyo, Japan) at a magnification of 100–15,000×.

The terminology of larvae follows that of Bohall et al. (1997), Hanelt and Janovy (2002), and Bolek et al. (2010).

### Phylogenetic analysis

Genomic DNA was extracted from fragments of horsehair worms and whole larvae using an ALS Tissue Genomic DNA Extraction Kit (Kaohsiung, Taiwan). A partial COI sequence was amplified by a polymerase chain reaction (PCR) with a set of universal primers (LCO1490 and HC02198) (Folmer et al. 1994). The PCR was initiated at 95°C for 5 min, followed by 35 cycles at 95°C for 1 min, 40°C for 1 min, and 72°C for 1 min, with a final extension at 72°C for 7 min. The PCR products were obtained by electrophoresis in 1.5% agarose gels and sequenced.

For the phylogenetic analysis, the COI sequence of *Paragordius* sp. (GenBank no. AY428843) was used as an outgroup. The 528 nucleotide base pairs of high quality were aligned using CLUSTALX 2.0.10 (Thompson et al*.* 1997). Pairwise genetic distances were calculated, and a phylogenic tree was reconstructed by the Neighbor-joining (NJ) method based on the Kimura 2-parameter model using MEGA 4.0.2 (Tamura et al. 2007). The support for the topology of the NJ tree was estimated by bootstrapping using 1000 replicates.

## Results

### Chordodes formosanus

Chiu, 2011 sp. n.

urn:lsid:zoobank.org:act:288E5D71-A694-4B65-BC15-164606F0DE4B

http://species-id.net/wiki/Chordodes_formosanus

#### Type locality.

Wufengqi Waterfalls (24°49'55.62"N, 121°44'50.10"E), Jiaushi Township, Yilan County, Taiwan (Holotype). Dachijieu (24°56'59.21"N, 121°34'2.12"E), Sindian (New Taipei City) (allotypes). Paratypes collected from Taiwan and Japan: Taipei Zoo (Taipei City), Sindian (New Taipei City), Taroko National Park (Hualien County), Wufengqi Waterfalls (Yilan County), Taiwan and Miyazaki Prefecture and Sakado (Saitama Prefecture), Japan. For detailed data, see Table 1.

#### Type material.

Partial bodies of holotype (male, 167 mm), and allotype (female, 282 mm) deposited at the Department of Entomology, National Taiwan University with the hosts. Paratypes deposited at the Department of Entomology, National Taiwan University, Taipei, and National Museum of Natural Science, Taichung, Taiwan and Lake Biwa Museum, Shiga, Japan. For detailed information, see Table 1.

#### Type-host.

*Hierodula formosana* Giglio-Tos (Mantodea: Mantidae). *Hierodula formosana* endemic to Taiwan, and the adult always emerging from late June to early August. Hosts of some samples belonging to *Hierodula patellifera* which distributed in both Taiwan and Japan. Their adults usually emerging in late autumn, about 2 months later than *Hierodula formosana*.

#### Etymology.

The specific name refers to Taiwan, the collection locality of the type specimens.

#### Description.

(Figs 1–5)

**Figure 1.**
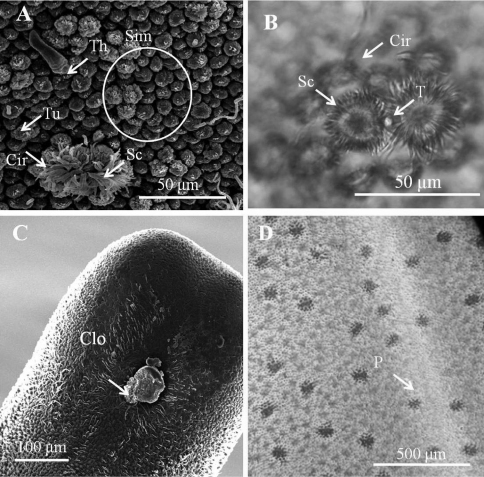
Male adult of *Chordodes formosanus* sp. n. **A** Cuticular surface with five types of areole **B** paired crowned areoles with a central tubercle **C** posterior end of the male **D** bright lengthwise regions with a darkly pigmented line on the ventral side of male body. Cir, circumcluster areole; Clo, cloacal opening; P, pigmented line; Sc, short-crowned areole; Sim, simple areoles; T, central tubercle; Th, thorn areole; Tu, tubercle areole.

**Figure 2.**
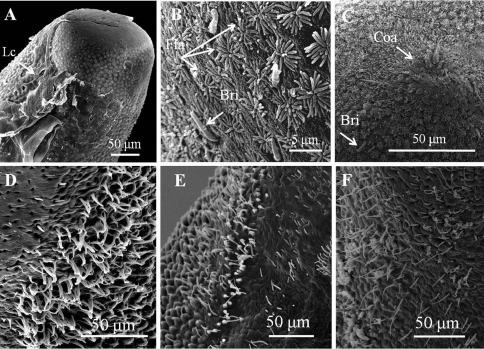
Details of ornamentations on anterior and posterior ends of male *Chordodes formosanus* sp. n. **A** Anterior end with larval cuticle **B** flat ornamentations and bristles on top of anterior end **C** cone-like areoles with bristles on top of anterior end **D–F** bristlefields with branched and unbranched bristles (D), short and unbranched bristles (E), or thin and unbranched bristles (F). Bri, bristle; Coa, cone-like areole; Lc, residual of larval cuticle; Fla, flat ornamentations.

*Male adult* (*n* = 17) (Figs 1, 2). Body length 74–277 mm, width (widest) 0.7–1 mm (after dehydration). In alcohol-preserved specimens, body rough and flat with dorsal and ventral grooves; dark-brown with bright lengthwise regions on both dorsal and ventral sides and darkly pigmented line on ventral side in most specimens (Fig. 1D).

Posterior end (Fig. 1C) not lobed, with short spines (ca. 5–12 μm) among areoles on margin. Cloacal opening subterminal, oval, 27–78 μm long and 17–63 μm wide. A pair of oval regions without areoles posterior to cloacal opening, each with scattered bristles extending as two rows of ventral strips (155–160 μm wide), structured by cord-like folds or flat areoles; flat areoles ornamented with short filaments in a cluster on top or scattered on cord-like folds, or absent. Paired oval bristlefields (70–77 μm wide and 145–243 μm long) bearing bristles on borders between flat areoles and normal areoles on lateral side of cloacal opening; bristles in bristlefields varying among individuals; some bearing only shorter or thinner unbranched bristles and some with both branched and unbranched bristles (Figs 2D–F). Anterior end tapered, same color as body, with white tip (white cap) but no dark collar under a stereomicroscope. Under SEM, anterior end round with moderately flat areoles and short bristles on surface; about 10 of them elevated and cone-like near anterior terminal; long thick bristles scattered among areoles, some between areoles and some penetrating areoles (Fig. 2C). Anterior end on one individual with residual larval cuticle tapered but flat terminally (Fig. 2A); also flat surrounding ornamentations and bristles (Fig. 2B). Mouth opens terminally in some individuals.

Entire body covered by areoles with cord-like folds in between. Areoles characterized into five types (simple, tubercle, thorn, circumcluster, and crowned areoles). Simple areoles (Fig. 1A), most abundant, covering most of body surface except anterior end and ventral side of posterior end; each 5–8 μm in diameter, more or less circular or oval, generally with a smooth surface but some with dots, grooves, or short bristles on surface. Simple areoles varying in height and some significantly elevated areoles in clusters of two to ten, looking like bulging areoles as mentioned by Schmidt-Rhaesa et al. (2008); but darker under light microscopy (Fig. 1D). Tubercle areoles (Fig. 1A) scattered among simple areoles, each shaped similarly to simple areole but with a tubercle (6–9 μm long) on apically concave center. Thorn areoles (Fig. 1A) distributed slightly along dorsal and ventral middle lines, similar to tubercle areoles but with a long solid thorn (22–57 μm long) instead of a tubercle. Thorn areoles small or absent in two samples. Crowned areoles clustered in pair with a central tubercle in between and surrounded by 12–20 circumcluster areoles with short filaments on apical surface (short-crowned areoles) (Figs 1A, B); scattered over trunk except anterior and posterior ends; each with medium filaments (10–15 μm) originating from apical center and sidelong to edges; only one male with a few crowned areoles containing a few filaments of around 100 μm.

**Figure 3.**
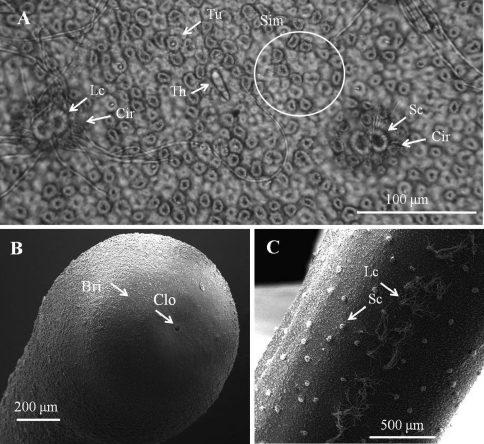
Female adult of *Chordodes formosanus* sp. n. **A** Cuticular surface with six types of areole **B** posterior end of female **C** ventral side of female body. Bri, bristle; Cir, circumcluster areole; Clo, cloacal opening; Lc, long crowned areole; Sc, short-crowned areole; Sim, simple areoles; Th, thorn areole; Tu, tubercle areole.

*Female adult* (*n* = 14) (Fig. 3). Length 263.7 (78–440) mm; body width (widest) 1–1.5 mm (after dehydration); body rough, flattened, dorsal and ventral grooves present; light to dark-brown with lengthwise regions on both dorsal and ventral sides, and darkly pigmented line on ventral side in most specimens. Some individuals with dark patches on bodies.

Posterior end (Fig. 3B) rounded, slightly swollen, covered by moderately flat areoles with cord-like folds surrounding cloacal opening; short bristles (10–27 μm) scattered between borders of moderately flat areoles and cord-like folds. Cloacal opening on terminal end, circular, 18–33 μm in diameter, no circumcloacal spine.

Anterior end with similar structure and color to males except lower cone-like areoles; terminally flat anterior end also appearing in one individual. Pattern and distribution of areoles (Fig. 3A) also similar to those of males but much more crowded in most individuals. Thorns of areoles shorter than those of most males (11–30 μm) but small or absent in three females. Cord-like folds present between areoles. Crowned areoles scattered over trunk as in males while roughly arranged in two lines on ventral and dorsal midlines, bearing significantly longer filaments (longest apical filaments ranging 65.57–392.25 μm (237.47 ± 66.22 μm, for details see “Diagnosis”)) (Figs 3A, C).

**Figure 4.**
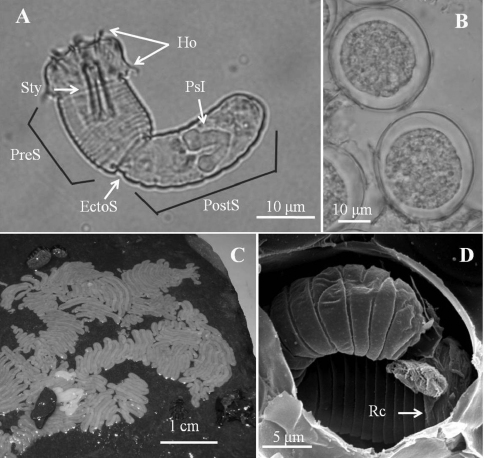
Eggs and larvae of *Chordodes formosanus* sp. n. **A** Live larva in water **B** eggs (6 days after being laid) **C** egg strings stuck onto a rock **D** larva in an egg with residual cuticle. EctoS, ectodermal septum; Ho, hooklet; PostS, postseptum; PreS, preseptum; PsI, pseudointestine gland; Rc, residual cuticle; Sty, stylet.

*Eggs* (Fig. 4).In laboratory, egg strings stuck onto substrate or drifting on bottom. Eggs (6 days after being laid) (Fig. 4B) nearly circular, 30.39 ± 1.15 μm (*n* = 10) in diameter. Egg strings white when laid and becoming light-brown within 1 day, turning dark-gray just before hatching. Eggs collected in field (Fig. 4C) all stuck onto rocks; mostly brown to gray, but some light-brown as those just laid in laboratory.

**Figure 5.**
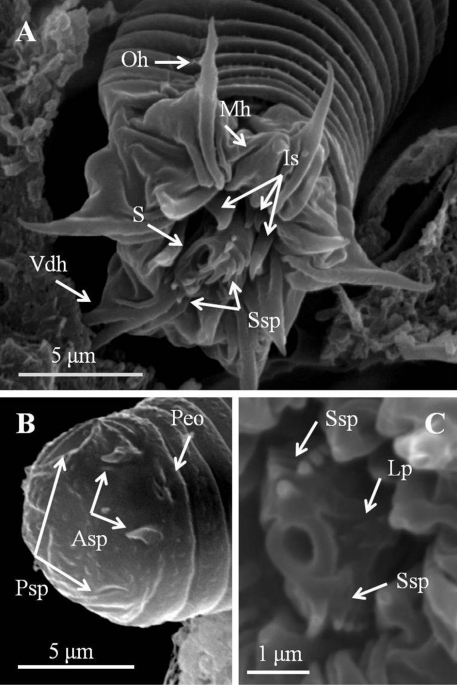
Detail of larvae of *Chordodes formosanus* sp. n. **A** Anterior view of a larva showing stylet and hook arrangement **B** posterior view of a larva **C** style with spines and lateral papillae. Asp, anterior terminal spine; Lp, lateral papillae; Is, inner spines; Mh, middle hook; Oh, outer hook; Peo, Pseudointestine exterior opening; Psp, posterior terminal spine; S, stylet; Ssp, stylet spines; Vdh, ventral double hook.

*Larvae* (Figs 4, 5). Larvae remaining near egg strings after hatching, not active. Under light microscopy, larval preseptum (Fig. 4A) averaging 20.55 (16.32–24.78) μm long and 13.21 (10.93–16.34) μm wide; postseptum averaging 24.91 (22.52–27.44) μm long and 10.06 (9.25–11.49) μm wide, stylet averaging 11.04 (9.59–13.25) μm long and 3.36 (2.76–3.91) μm wide. Pseudointestines V-shaped (Fig. 4A) with one small and one large branch, both with a swelling on posterior ends. Large branch averaging 8.27 (7.28–9.82) μm, small branch averaging 6.70 (5.43–7.59) μm long. Under SEM, larvae superficially annulated with 13 segments on preseptum and 10 on postseptum, ectodermal septum as a single segment between them. Three sets of hooks arranged in three rings on anterior preseptum (Fig. 5A): outer ring containing seven hooks (outer hooks), two ventrally positioned and closely together on base (ventral double hook); six hooks on second ring located between each outer hook (middle hook); inner ring containing at least three inner spines, but real number unknown. A stylet (Figs 5A, C) appearing inside preseptum, ornamented with two sets of spines: nine spines on dorsal and ventral sides of stylet, five small lateral papillae on left side. A pair of anterior and posterior terminal spines (Fig. 5B) on posterior of postseptum. Pseudointestine exterior opening (Fig. 5B) centrally located between anterior terminal spines on ventral body. Several larvae covered by residual skin: one observed in broken egg suggesting that molting had occurred before emergence (Fig. 4D).

#### Diagnosis.

Horsehair worms from the mantids *Hierodula formosana* and *Hierodula patellifera* were characterized by all six types of areoles, including simple, tubercle, thorn, circumcluster, short-crowned, and long-crowned areoles in the female. The same six areole types are similar to those of *Chordodes japonensis* described by Inoue (1952) and Baek (1993). Nevertheless, the significantly longer filaments on female crowned areoles suggest they belong to a new species, *Chordodes formosanus* sp. n. By the way, the absence of long-crowned areoles in our male sample of *Chordodes formosanus* sp. n. probably implies their potential for distinguishing these two different species. However, since the dimorphism of male crowned areoles has not been mentioned in *Chordodes japonensis*, more studies are needed to uncover this phenomenon.

The crowned areole is an autapomorphy of the genus *Chordodes*. In *Chordodes formosanus* sp. n. and *Chordodes japonensis*, it is composed of two major areoles ornamented with apical filaments and several surrounding circumcluster areoles. The dimorphic length of the apical filaments divides the crowned areoles into two types, short-crowned areoles with short ornamental filaments and long-crowned areoles with long ones. All samples we checked (both sexes of *Chordodes formosanus* sp. n. and one male *Chordodes japonensis*) had short-crowned areoles scattered all over the body trunk, with the long-crowned areoles only appearing on the ventral and dorsal midlines of the female *Chordodes formosanus* sp. n. andmale *Chordodes japonensis*, but not themale *Chordodes formosanus* sp. n. We did not personally observe the female *Chordodes japonensis*, but these dimorphic crowned areoles must be present according to the descriptions of Inoue (1952) and Baek (1993). Additionally, the apical filament lengths of long-crowned areoles were significantly longer on *Chordodes formosanus* sp. n. We randomly chose two to five sets of long-crowned areoles from our female samples and measured each of their longest apical filaments. In the 68 sets of long crowned areoles, the longest apical filaments ranged 65.57–392.25 (237.47 ± 66.22) μm. Fifty-one of these 68 (75%) sets of crowned areoles had apical filaments of > 200 μm. The longest apical filaments in our male *Chordodes japonensis* were 92.03–139.70 μm. Significantly shorter filaments in *Chordodes japonensis* were also described by Inoue (1952) and Baek (1993). *Chordodes japonensis* has long-crowned areoles with filaments of around 50 μm (Inoue 1952; fig. 1a) and filaments of < 200 μm in the description by Baek (1993). For other differences and detailed comparisons, see Table 2 and the “Discussion”.

**Table 2.** Comparison of areolar types between *Chordodes formosanus* sp. n. (Schmidt-Rhaesa 2004, samples of which were considered to be *Chordodes japonensis*), *Chordodes japonensis* (Inoue 1952, Baek 1993) and in this investigation

Areolar type	*Chordodes formosanus* sp. n.		*Chordodes japonensis*		
	This study	Schmidt-Rhaesa (2004)	This study	Inoue (1952)	Baek (1993)
Sample size	17 ♂♂, 22 ♀♀	1 ♂**, 1 ♀	1 ♂	49 ♂♂, 37 ♀♀	17 ♂♂, 7 ♀♀
Collecting locality	Taiwan, Japan	Japan	Japan	Japan	Korea
Host	*Hierodula formosana* and *Hierodula patellifera*	*Hierodula patellifera* (unknown for the male worm)	*Tenodera sinensis*	Not mentioned	Not mentioned
Crowned areoles with short projections	+	+	+	-	+
Crowned areoles with long projections	+ (most areoles with filaments > 200 μm)	+ (> 200 μm)	+ (mostly around 100 μm, maximum 140 μm)	+ (around 50 μm in Fig. 1A)	+ (< 200 μm)
Sexual dimorphism in crowned areoles	+	-	? (Only males were investigated)	-	-
Circumcluster areoles	+ (12-20)	+ (at least 12 in Fig. 2C)	+ (7-14)	+ (10 in Fig. 1A)	+ (7-17)
Tubercle areoles	+	+	+	+	+
Thorn areoles (spine areoles)	+ (small or absent in some samples; 9/40)	-	+	+	-
Bulging areoles	* (some elevated simple areoles in clusters of 2-10)	- (simple areoles elevated differently in Fig. 2A and 2D)	* (some elevated simple areoles in clusters of 2-5)	- (paired elevated areoles)	- (simple areoles elevated differently in Fig1)
Simple areoles	+	+	+	+	+

Terminology based on Schmidt-Rhaesa (2004) and Schmidt-Rhaesa et al. (2008). +, present; -, absent; *, difficult to determine; ?, unknown. **, According to the description, we consider the specimen not to be *Chordodes formosanus* sp. n.

### Chordodes japonensis

Inoue, 1952

http://species-id.net/wiki/Chordodes_japonensis

#### Material examined.

Examined male collected with its host from Miyazaki Prefecture, Japan (32°10'21.50"N, 131°27'36.53"E) by Yasukuni Ono on 5-XI-2010. Partial body of horsehair worm deposited with its host at Department of Entomology, National Taiwan University, Taipei, Taiwan. Accession number of partial COI sequence in GenBank: JF808206.

#### Host.

Chinese mantids, *Tenodera sinensis* (Mantodea: Mantidae), which are sometimes classified as *Tenodera aridifolia*.

#### Redescription.

(Fig. 6)

*Male adult* (*n* = 1). Body length 220 mm, width (widest) 0.94 mm (after dehydration). In alcohol-preserved specimens, body rough and flat with dorsal and ventral grooves, dark-brown with a darkly pigmented line on ventral side.

**Figure 6.**
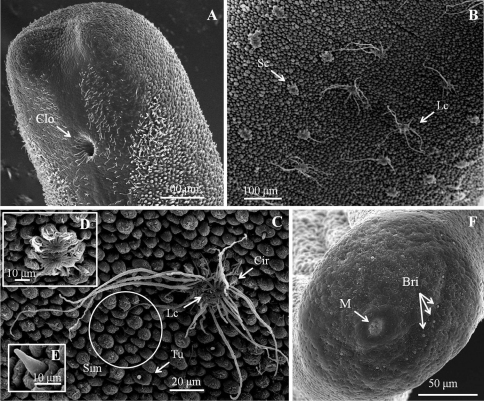
Male adult of *Chordodes japonensis*. **A** Posterior end of male **B** ventral side of male body **C** cuticular surface with four types of areole **D** short-crowned areoles **E** thorn areole **F** anterior end. Bri, bristle; Cir, circumcluster areole; Clo, cloacal opening; Lc, long crowned areole; M, mouth; Sc, short-crowned areole; Sim, simple areoles; Tu, tubercle areole.

Posterior end (Fig. 6A) not lobed, with short spines (ca. 5–13 μm) among areoles on margin. Cloacal opening subterminal, oval 44 μm long and 25 μm wide, with circumcloacal spines. A pair of oval regions without areoles posterior to cloacal opening, each with scattered bristles extending as two rows of ventral strips (115–120 μm wide) structured by cord-like folds or flat areoles. Paired oval bristlefields (82 μm wide and 231 μm long) bearing numerous branched and unbranched bristles on borders between flat areoles and normal areoles on lateral side of cloacal opening. Anterior end tapered, same color as body, with white tip (white cap) but no dark collar under stereomicroscopy. Under SEM, anterior end (Fig. 6F) smooth with short, thick bristles and small spines; mouth open on cone at anterior extremity.

Entire body covered by areoles with slightly cord-like folds in between. Areoles characterized into six types (simple, tubercle, thorn, circumcluster, and two types of crowned areoles). Simple areoles (Fig. 6C), most abundant, covering entire body surface except anterior end and ventral side of posterior end; each 5–11 μm in diameter, more or less circular or oval, surface uneven, some areas with short bristles. Simple areoles varying in height and some significantly elevated areoles in clusters of two to five, appearing as bulging areoles but darker under light microscopy. Tubercle areoles (Fig. 6C) scattered among simple areoles, each similarly shaped to simple areole but with a tubercle (about 7 μm long) on apically concave center. Thorn areoles (Fig. 6E) similar to tubercle areoles but with a long solid thorn (15 μm long) instead of tubercle; number of thorn areoles much fewer than tubercle areoles. Crowned areoles (Figs 6B, D) clustered in pairs with a central tubercle in between and surrounded by 7–14 circumcluster areoles with short filaments on apical surface; scattered over trunk; each with medium filaments (12–20 μm) originating from apical center and sidelong to edges. Crowned areoles roughly arranged in two lines on ventral and dorsal midlines, bearing significantly longer filaments (most around 100 μm, none > 150 μm) (Figs 6B, C).

#### Phylogeny.

**Figure 7.**
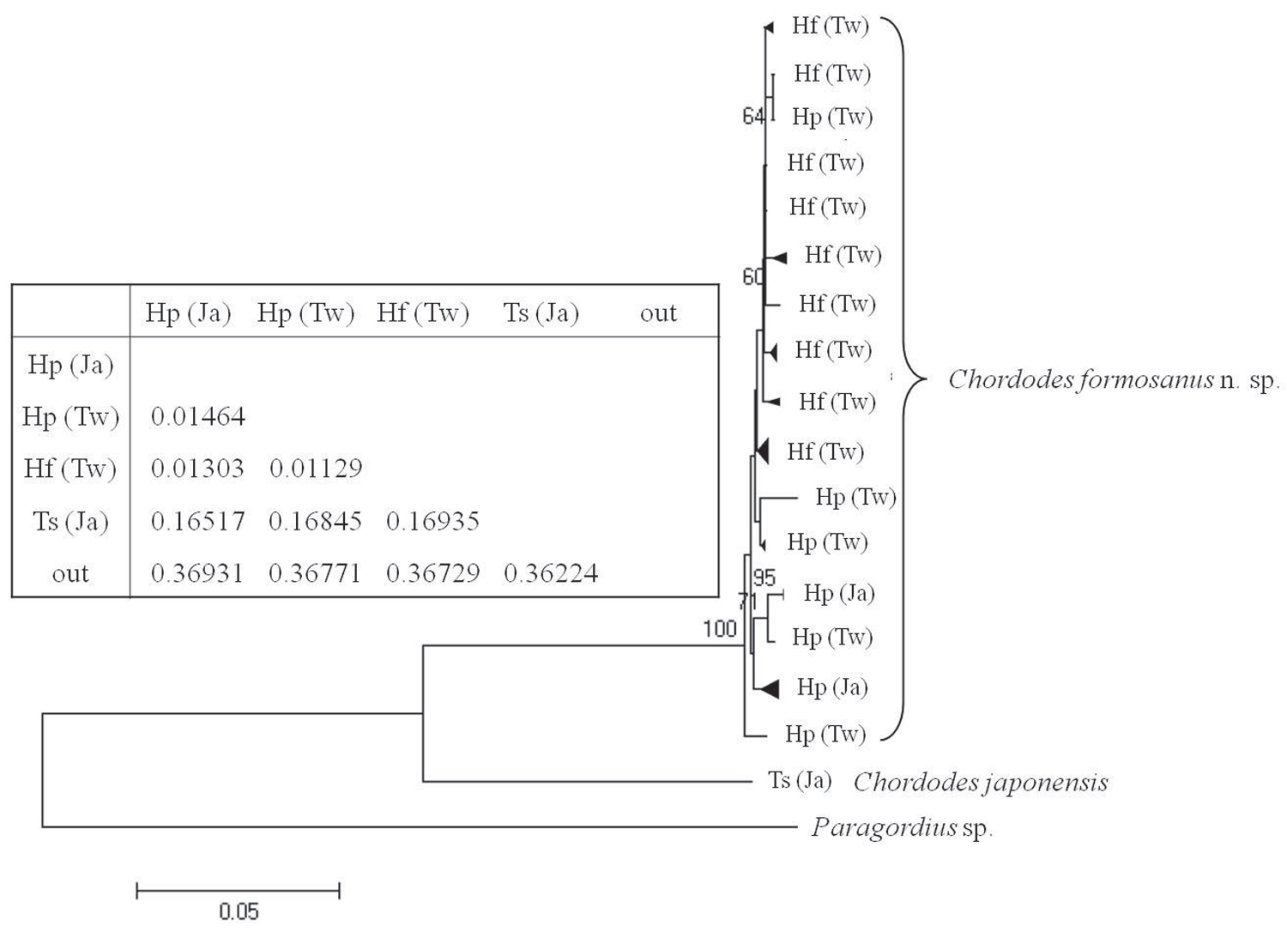
Neighbor-joining tree of *Chordodes formosanus* sp. n. and *Chordodes japonensis* with genetic distances between each group. Abbreviations in the table indicate the horsehair worms’ mantid hosts (Hf, *Hierodula formosana*; Hp, *Hierodula patellifera*; Ts, *Tenodera sinensis*) and collecting localities (Tw, Taiwan; Ja, Japan). Numbers at the nodes represent the percentage of 1000 bootstrap values of > 70%. The outgroup (out) was a *Paragordius* sp.

A phylogenetic tree of the 40 samples of horsehair worms collected from three species of mantids *Hierodula formosana*, *Hierodula patellifera*, and *Tenodera sinensis* from Taiwan and Japan with one outgroup (*Paragordius* sp.) is shown in Fig. 7.

Comparison of the 40 horsehair worm samples (39 *Chordodes formosanus* and one *Chordodes japonensis*) revealed there were 31 haplotypes with 432 invariable sites, 64 singletons, and 32 parsimoniously informative sites. Newly sequenced COI data were deposited in the GenBank database (see Table 1 for accession nos.). Samples from the host mantids of *Hierodula* and *Tenodera* (which are considered two species, *Chordodes formosanus* sp. n. and *Chordodes japonensis*, respectively)were significantly separated into two groups based on the phylogenetic tree restructured by the NJ method (Fig. 7). The genetic distance between these two groups was 0.16840. This result supports that theybelong to different species as we suggested based on morphology. The phylogenetic tree also revealed a polytomic topology among the 39 horsehair worms parasitizing *Hierodula*. Although some clades were observed, they were not highly supported due to low bootstrap values and short genetic distances; the mean genetic distances among them was 0.00979 with a range of 0.000-0.01922. Since the genetic distance between the larvae collected in the field and *Chordodes formosanus* was only 0.00759, we suggest that those larvae belong to the same species.

#### Discussion.

In this article, a new species, *Chordodes formosanus* sp. n., which parasitizes *Hierodula formosana* and *Hierodula patellifera*, was proposed and described including the morphology of the egg and larval stages. Because of a similar morphological description by Schmidt-Rhaesa (2004), we categorized the species in that study into *Chordodes formosanus* sp. n. and also limited the mantid host range of *Chordodes japonensis* to the genus *Tenodera*.

##### Morphological comparison of *Chordodes formosanus* sp. n. and *Chordodes japonensis*

The two species, *Chordodes formosanus* sp. n. and *Chordodes japonensis*, can be distinguished by the presence of dimorphism in male crowned areoles and the length of long filaments on female crowned areoles. A comparison of areolar types in *Chordodes formosanus* sp. n. and *Chordodes japonensis* is given in Table 2. Both *Chordodes formosanus* sp. n. and *Chordodes japonensis* have two forms of crowned areoles on their cuticles, one with moderate attachments on the top (short-crowned areoles) and the other with significantly longer attachments (long-crowned areoles). Both types of crowned areoles were found on these two species except for the male *Chordodes formosanus* sp. n. (the short-crowned areoles were not mentioned in the description of *Chordodes japonensis* by Inoue (1952)), which indicates sexual dimorphism in *Chordodes formosanus* sp. n. We believe the female worm which was previously considered to be *Chordodes japonensis* by Schmidt-Rhaesa (2004) actually belongs to the species, *Chordodes formosanus* n. sp, described here. The reason why the sexual dimorphism was not mentioned by Schmidt-Rhaesa (2004) is probably that the free-living male is not the same species as the female. In addition, the length of the filaments on the long-crowned areoles is also a significant character differentiating these two species; they are always > 200 μm in *Chordodes formosanus* sp. n. (or > 6-fold that of paired crowned areoles) but < 200 μm in *Chordodes japonensis* (or < 5-fold that of paired crowned areoles, and most of them are around 100 μm).

In addition to the crowned areoles, other minor differences in Table 2 (thorn areoles and bulging areoles) are much more difficult to use in discriminating these two species. Although the presence of thorn areoles is always questionable due to their small number, it is still considered a key character for distinguishing different species (Schmidt-Rhaesa et al. 2008). Thorn areoles were not reported by Schmidt-Rhaesa (2004) or Baek (1993) but appeared in the description by Inoue (1952). In our study, thorn areoles were not found in nine *Chordodes formosanus* sp. n. but appeared in the other 31 *Chordodes formosanus* sp. n. and one *Chordodes japonensis*. Contrary to the thorn areoles which can be easily distinguished from other types of areoles, bulging areoles are much easier to be confused with simple areoles (Schmidt-Rhaesa et al. 2008). Although bulging areoles were consistently ignored in descriptions of *Chordodes japonensis*, we observed various heights of simple areoles, and some of them clustered in groups similar to bulging areoles as described by Schmidt-Rhaesa et al. (2008). This renders the presence of bulging areoles in *Chordodes japonensis* as well as *Chordodes formosanus* sp. n. questionable.

##### Molting and environmental effects on morphology

About 90 species belong to the genus *Chordodes* which were proposed using only five characteristic types of areoles (Schmidt-Rhaesa et al. 2008). Unfortunately, most characters other than these areole types have seldom been mentioned. According to our observations, the complex ornamentations of the structure of the anterior end were previously considered to be smooth. A horsehair worm explores unknown environments with its head, and the head is also the first part which contacts outer environments when they emerge from a host. Schmidt-Rhaesa and Gerke (2006) suggested that the apical filaments of the crowned areoles may have sensory functions. It is rational that the anterior and posterior ends of horsehair worms with the complex ornamentations play important roles in exploration and mating. Therefore, the smooth anterior ends in some descriptions (e.g., de Villalobos and Zanca 2001) and some of our samples may have been caused by damage from the environment. In addition to the damage, some samples were found to have been covered by residual juvenile skins, indicating that the worms had just molted. Shapes of the ornamentations on the ends under the larval cuticle were flat. The molting of hairworms was observed (Schmidt-Rhaesa et al. 2003), and its potential effects on the morphology should be carefully considered in the future studies. Residual skin was also found on newly hatched larvae, and on a larva still inside an egg, indicating that molting occurred before it emerged from the egg. As a group of Ecdysozoa (Aguinaldo et al. 1997), horsehair worms molt between each instar. According to our observations of metamorphosis from cysts to wormlike juveniles and from juveniles to adults, we suspect those horsehair worms may proceed through at least three molts before maturing.

##### Host specificity

Compared to the wide range of paratenic hosts, horsehair worms’ definitive host range is limited to one or a few species. Because nematomorphs are sometimes found after they have emerged from their hosts, definitive information on hosts is unknown in some species. *Chordodes japonensis* was reported to be parasites of the mantids, *Tenodera sinensis*, *Tenodera angustipennis* (Inoue 1952), *Hierodula patellifera* (Schmidt-Rhaesa 2004), and longhorn grasshoppers, *Hexacentrus japonicus japonicus* (Inoue 1955) in Japan. Since the hairworm described by Schmidt-Rhaesa (2004) was here considered to be *Chordodes formosanus* sp. n., the mantid host of *Chordodes japonensis* is now limited to two species of *Tenodera*. Until now, we have no evidence to exclude *Hexacentrus japonicus japonicus* from being a potential host of *Chordodes japonensis*. However, in our investigation, the host range of these two species seems to be limited to the generic level. Therefore, we still question the ability of *Chordodes japonensis* to develop inside hosts other than mantids.

##### Molecular approach and perspectives

In the 40 samples from Taiwan and Japan we examined, the taxonomic status was supported not only by their morphologies, but also by the partial COI sequences. COI sequences were used to study inter- and intraspecific relationships due to the high mutation rate (Hebert et al. 2003). The low genetic divergence among our hairworm samples suggests their conspecific relationship, and our samples can also be separated from those hairworms that emerged from *Tenodera* by theirsignificantly divergent sequences. Since species of immature stages can only be conjectured by adults in the same area (Winterbourn 2005), molecular data were herein proven to be a useful tool for identifying both adults and immatures. As molecular information for the phylum Nematomorpha is still limited, we believe more molecular data would be helpful and can be used to uncover uncertain relationships among horsehair worms in the near future.

## Supplementary Material

XML Treatment for Chordodes formosanus

XML Treatment for Chordodes japonensis
